# Consultation with health professionals for mental health in Australia in 2020–2022 and changes since 2007: Findings from the 2020–2022 National Study of Mental Health and Wellbeing

**DOI:** 10.1177/00048674241307919

**Published:** 2025-07-15

**Authors:** Meredith G Harris, Caley Tapp, Joshua J Vescovi, Matthew Sunderland, Sandra Diminic, Cath Chapman, Tim N Slade, Maree Teesson, Jane Pirkis, Philip M Burgess

**Affiliations:** 1 School of Public Health, The University of Queensland, Brisbane, QLD, Australia; 2 Queensland Centre for Mental Health Research, Brisbane, QLD, Australia; 3 The Matilda Centre for Research in Mental Health and Substance Use, The University of Sydney, Sydney, NSW, Australia; 4 Centre for Mental Health, The University of Melbourne, Melbourne, VIC, Australia

**Keywords:** Consultation with health professionals, mental disorders, substance use disorders, epidemiology, survey, health policy

## Abstract

**Objective:**

This study aimed to estimate the proportions of Australians aged 16–85 years who consulted health professionals for mental health in 2020–2022, and changes since 2007.

**Methods:**

Secondary analysis of merged data from the National Study of Mental Health and Wellbeing in 2020–2022 (*N* = 15,893) and its 2007 predecessor (*N* = 8841).

**Results:**

In 2020–2022, 17.4% of Australians aged 16–85 years had consulted a health professional (including overnight hospital admission) for their mental health in the past year (vs 11.9% in 2007). The largest increases between the surveys were in use of psychologists (123%), other (non-medical) mental health professionals (64%), and general practitioners (53%). Of adults with a 12-month mental disorder, 46.6% consulted a health professional in 2020–2022 (vs 37.5% in 2007), increasing with severity (mild 22.9%, moderate 48.4% and severe 68.8%). Multivariate regression models showed that consulting a health professional was positively associated with age < 65 years, female sex, being unmarried, disorder severity, and affective or anxiety disorder; these patterns held for consultations with most types of professionals. Socioeconomic and geographical characteristics were associated with consulting particular professionals: lower income (consulting a psychiatrist), high income and living in a major city (psychologist), living in a less disadvantaged area (general practitioner). Increases in consulting were not experienced by all groups (e.g. the likelihood of consulting increased for people aged 16–45 years, but not for older age groups).

**Conclusion:**

Consultation with health professionals for mental health improved between 2007 and 2020–2022 but remains below national targets. Some changes may reflect recent service reforms; however, gaps in access persist.

## Introduction

Australia spends up to US$16 billion annually on mental health-related services (AIHW, 2023a; Productivity Commission, 2020). However, service gaps and systemic barriers mean that many people with mental health needs do not access care (Burgess et al., 2009). Improving access requires an understanding of how mental health care is distributed in the community, and how these patterns change over time. This information can be obtained from repeated administrations of national population surveys.

Reports from Australia’s second National Survey of Mental Health and Wellbeing (NSMHWB) (Slade et al., 2009) showed that, in 2007, just over one-third of adults with 12-month affective, anxiety, and substance use disorders had consulted a health professional for their mental health in the previous year, and only two-thirds with severe disorders had done so. These proportions were little changed since the first survey in 1997 (Burgess et al., 2009). The use of psychologists doubled between 1997 and 2007 – a likely impact of primary mental health care reforms implemented during that period – but favoured higher-income earners (Bartram and Stewart, 2019; Burgess et al., 2009). People with substance use disorders, males, and younger and older adults were underrepresented among people who consulted a health professional for mental health (Chapman et al., 2015; Gonçalves et al., 2014; Reavley et al., 2010; Teesson et al., 2009). People without a 12-month mental disorder, but with other potential indicators of treatment need (e.g. prior disorders, subthreshold symptoms), consulted a similar mix of providers but had fewer visits compared to those with disorders (Bobevski et al., 2017; Harris et al., 2014).

Since 2007, there have been important changes to Australia’s mental health service offerings. These include the implementation of stepped care at a local level through Primary Health Networks; the growth of primary mental health care programmes (e.g. headspace youth services and Medicare-subsidised services through the Better Access programme); increased capacity and accessibility of digital crisis support services (e.g. Beyond Blue and Kids Helpline); new digital services offering mental health information and support (e.g. through Head to Health); new low-intensity psychological therapy services (e.g. NewAccess); and new services for people with severe mental health problems (e.g. psychosocial disability support services through the National Disability Insurance Scheme [NDIS] and clinical services through Adult Mental Health Centres) and people with complex mental health needs (e.g. SANE’s Guided Service) (Department of Health, 2019; Department of Health and Aged Care, 2022a, 2022b, 2023a, 2023b; National Disability Insurance Agency, 2023; SANE, n.d.).

A third survey, the 2020–2022 National Study of Mental Health and Wellbeing (NSMHWB), provided an opportunity to obtain updated estimates of consultation with health professionals for mental health and to explore changes since 2007 across clinical and sociodemographic subgroups.

## Method

### Design

We undertook a secondary analysis of data from the 2020–2022 and 2007 NSMHWBs. The NSMHWBs were epidemiological surveys of the mental health of Australian adults funded by the Australian Government and carried out by the Australian Bureau of Statistics (ABS). The 2020–2022 and 2007 survey design and methods are described and compared in detail elsewhere (ABS, 2008, 2023a). To summarise, the samples represent all usual residents aged 16–85 years living in private dwellings in urban and rural areas across all states and territories of Australia. Very remote areas (both surveys) and discrete First Nations communities (2020–2022 survey) were excluded. Households were randomly selected, and one eligible resident from each household was randomly selected to complete an interview. People aged 16–24 years (both surveys) and 65–85 years (2007 survey) were oversampled to improve the reliability of estimates for these groups. The 2020–2022 survey sample was recruited in two cohorts due to COVID-19-related lockdowns (Cohort 1: *n* = 5554 recruited December 2020–July 2021, response rate 57.1%; Cohort 2: *n* = 10,339 recruited December 2021–October 2022, response rate 49.6%). The ABS created a combined sample of 15,893 respondents (overall response rate 52.0%). The 2007 survey sample included 8841 respondents (response rate = 60%) recruited in a single cohort (August–December 2007). Interviews were conducted face-to-face by ABS interviewers in both surveys, excepting for a small number (*n* = 447) in Cohort 2 of the 2020–2022 NSMHWB that were conducted by video call due to lockdowns. The current study was deemed exempt from human research ethics review by Research Ethics and Integrity, The University of Queensland (Project no. 2022/HE001538).

### Survey instrument

The World Mental Health Survey Initiative version of the World Health Organisation’s (WHO) Composite International Diagnostic Interview, version 3.0 (WMH-CIDI 3.0; Kessler and Ustün, 2004), a fully structured diagnostic interview, was used to assess lifetime mental disorders in three classes: affective (mood) disorders; anxiety disorders; and substance use disorders. WMH-CIDI 3.0 algorithms generated diagnoses according to *Diagnostic and Statistical Manual of Mental Disorders*, Fourth Edition (*DSM*-IV). Questions about symptoms experienced during the previous 12 months were combined with lifetime disorder information to determine 12-month disorder prevalence. Among those with any 12-month disorder, a level of severity (mild, moderate or severe) was determined from responses relating to disorder-specific role impairment and other clinical factors (*DSM*-IV Australian version).

Consultations with health professionals for mental health were assessed in a service utilisation module designed for the Australian context. Respondents were asked about overnight hospital admissions relating to their mental health problems (including but not restricted to stress, anxiety, depression or dependence on alcohol or drugs) and consultations with different types of health professionals for their mental health in the previous 12 months. In the 2020–2022 survey, the types of health professionals were general practitioners (GPs), psychiatrists, psychologists, mental health nurses, other mental health professionals (including social workers, counsellors or occupational therapists), specialist doctors or surgeons (including cardiologists, gynaecologists or urologists) and other health professionals (including dietitians, physiotherapists or pharmacists). Most categories were the same in the 2007 survey, except that ‘other mental health professionals’ was worded ‘other professional providing specialist mental health services’, and ‘other health professionals’ was worded ‘other professional providing general services’ with the examples ‘social worker, occupational therapist, counsellor’. In addition, the 2007 survey had a separate category for ‘complementary/alternative therapist, such as a herbalist or naturopath’; in 2020–2022, respondents might have included these in the ‘other mental health professional’ or ‘other health professional’ categories (ABS, 2023a). For this study, we included mental health nurses in the ‘other mental health professionals’ category and included specialist doctors or surgeons, and complementary/alternative therapists (2007 only) in the ‘other health professionals’ category.

### Statistical analysis

The 2020–2022 and 2007 survey datasets were merged to allow comparisons over time (Moser et al., 2013). Building on previous analyses by members of our team (Burgess et al., 2009), we first estimated proportions consulting health professionals for mental health and adjusted Wilson 95% confidence intervals (CIs) (Dean and Pagano, 2015) separately for each survey year. We then explored associations of time (survey year) and respondent characteristics with consultation with health professionals overall and for each category (except for overnight hospitalisations, due to the small numbers) using multivariable logistic regression models. Interactions between survey year and respondent characteristics were estimated, and subgroup analysis was performed to quantify the changes over time for subgroups. All weighted calculations were made using the survey weights provided by the ABS (2008, 2023a). Data were analysed using the ‘Survey’ package (Lumley, 2023) within R Statistical Software (v4.3.0) (R Core Team, 2023) via the ABS’s secure DataLab environment.

## Results

### Consultation with health professionals for mental health

Table 1 shows that, in 2020–2022, approximately one in every six Australian adults (17.4%, 95% CI = [16.6, 18.2]) had consulted a health professional for their mental health in the past year. Relatively more people consulted GPs (12.4%, 95% CI = [11.7, 13.1]) than any other type of professional, followed by psychologists (7.8%, 95% CI = [7.3, 8.3]). Relatively few people had overnight hospital admissions (0.6%, 95% CI = [0.5, 0.8]). Females consulted a health professional, and consulted GPs, psychologists, and other mental health professionals, more commonly than males.

**Table 1.** Consultation with health professionals for mental health in the past 12 months, by sex (2020–2022).

	Males			Females			Persons		
	EPC (‘000)	%	95% CI	EPC (‘000)	%	95% CI	EPC (‘000)	%	95% CI
Consultation with any health professional for mental health^a^	1264.1	13.0	[11.9, 14.1]	2176.4	21.6	[20.6, 22.7]	3440.6	17.4	[16.6, 18.2]
General practitioner	825.0	8.5	[7.6, 9.4]	1623.9	16.2	[15.2, 17.2]	2449.0	12.4	[11.7, 13.1]
Psychiatrist	297.2	3.0	[2.6, 3.6]	355.9	3.5	[3.1, 4.0]	653.1	3.3	[3.0, 3.6]
Psychologist	555.1	5.7	[5.1, 6.4]	983.3	9.8	[9.1, 10.5]	1538.4	7.8	[7.3, 8.3]
Other mental health professional^b^	236.0	2.4	[2.0, 2.9]	475.6	4.7	[4.1, 5.4]	711.6	3.6	[3.2, 4.0]
Other health professional^c^	82.8	0.8	[0.6, 1.2]	145.3	1.4	[1.2, 1.8]	228.1	1.2	[1.0, 1.4]
Overnight hospital admission	52.1	0.5	[0.4, 0.7]	65.0	0.6	[0.5, 0.9]	117.1	0.6	[0.5, 0.8]
No consultation with a health professional for mental health	8492.7	87.0	[85.9, 88.1]	7881.6	78.4	[77.3, 79.4]	16,374.3	82.6	[81.8, 83.4]

CI: confidence interval. EPC: estimated population count. The 2020-22 sample for analysis excluded persons who described their sex at birth as Another term (n=15,891). All estimates exclude respondents with missing data, which ranged from 0.00% to 0.15% (unweighted) per estimate.

a Includes consultation with a general practitioner, psychiatrist, psychologist, other mental health professional or other health professional for mental health, or overnight hospital admission for mental health problems.

b Includes mental health nurse; and other mental health professionals (including social worker, counsellor or occupational therapist).

c Includes other health professional (including dietitian, physiotherapist or pharmacist) and specialist doctor or surgeon (including cardiologist, gynaecologist or urologist).

The proportion of people who consulted a health professional for their mental health increased by 46% between 2007 and 2020–2022 (see Supplementary Table S1 for 2007 survey estimates), with the largest changes for psychologists (123%), other mental health professionals (64%), GPs (53%) and other health professionals (−50%), noting that the examples of professionals included in the latter category differed between the surveys.

### Consultation with health professionals for mental health among people with disorders

Tables 2 and 3 show that in 2020–2022, nearly half (46.6%, 95% CI = [44.2, 48.9]) of people with 12-month affective, anxiety or substance use disorders had consulted a health professional for their mental health. Conversely, more than half (53.4%, 95% CI = [51.1, 55.8]) had not done so. Males consulted (38.6%, 95% CI = [35.0, 42.3]) less commonly than females (52.4%, 95% CI = [49.4, 55.3]); in particular, males aged 16–24 and 35–44 years consulted less commonly than same-age females. People aged 55–85 years consulted less commonly than those aged 25–44 years; females aged 55–85 years consulted less commonly than females aged 35–44 years.

**Table 2.** Consultation with health professionals for mental health in the past 12 months among people with any 12-month disorder, by age group and sex (2020–2022).

Age group (years)	Males			Females			Persons		
	EPC (‘000)	%	95% CI	EPC (‘000)	%	95% CI	EPC (‘000)	%	95% CI
16–24	143.0	34.2	[28.5, 40.4]	312.4	55.8	[49.6, 61.8]	455.2	46.5	[42.0, 51.1]
25–34	172.9	43.7	[36.0, 51.8]	293.9	56.3	[51.1, 61.3]	466.8	50.9	[46.5, 55.2]
35–44	120.2	43.3	[35.9, 51.1]	257.5	61.4	[54.9, 67.5]	377.9	54.2	[49.3, 59.1]
45–54	81.9	34.6	[25.3, 45.2]	156.0	46.4	[37.2, 55.8]	237.9	41.5	[35.0, 48.3]
55–64	85.6	40.7	[29.5, 52.8]	109.5	39.1	[31.7, 47.0]	195.1	39.8	[34.3, 45.5]
65–85	48.0	32.0	[22.8, 42.7]	87.2	42.5	[34.3, 51.2]	135.2	38.1	[32.1, 44.5]
Total	651.7	38.6	[35.0, 42.3]	1216.5	52.4	[49.4, 55.3]	1868.3	46.6	[44.2, 48.9]

CI: confidence interval. EPC: estimated population count. All estimates exclude respondents with missing data, which ranged from 0.00% to 0.52% (unweighted) per estimate. Consultation with health professionals includes consultation with a general practitioner, psychiatrist, psychologist, other mental health professional or other health professional for mental health, or overnight hospital admission for mental health problems. Table includes persons who met criteria for diagnosis of a DSM-IV lifetime affective, anxiety or substance use disorder (with hierarchy) assessed in the survey and had sufficient symptoms of that disorder in the 12 months prior to interview.

**Table 3.** Consultation with health professionals for mental health in the past 12 months, by 12-month disorder class (2020–2022).

	Any 12-month affective disorder		Any 12-month anxiety disorder		Any 12-month substance use disorder		Any 12-month disorder ^a^		No 12-month disorder ^b^	
	EPC (‘000) = 1472.7		EPC (‘000) = 3119.1		EPC (‘000) = 622.4		EPC (‘000) = 4011.9		EPC (‘000) = 15812.9	
	%	95% CI	%	95% CI	%	95% CI	%	95% CI	%	95% CI
Consultation with any health professional for mental health^c^	63.7	[60.3, 67.0]	48.0	[45.2, 50.8]	41.9	[36.6, 47.4]	46.6	[44.2, 48.9]	10.0	[9.3, 10.7]
General practitioner	52.7	[49.3, 56.0]	38.6	[36.0, 41.3]	32.8	[27.7, 38.3]	37.2	[35.0, 39.4]	6.1	[5.6, 6.7]
Psychiatrist	16.1	[14.0, 18.5]	10.7	[9.4, 12.1]	10.6	[7.3, 15.1]	10.3	[9.2, 11.6]	1.5	[1.2, 1.9]
Psychologist	29.7	[26.5, 33.0]	23.3	[21.5, 25.2]	17.7	[13.7, 22.6]	21.6	[20.0, 23.4]	4.2	[3.8, 4.7]
Other mental health professional^d^	15.5	[12.7, 18.8]	11.1	[9.6, 12.8]	13.3	[9.7, 18.1]	10.3	[9.1, 11.7]	1.9	[1.6, 2.3]
Other health professional^e^	5.1	[3.6, 7.3]	4.0	[3.2, 5.0]	2.5	[1.1, 5.4]	3.5	[2.8, 4.4]	0.5	[0.4, 0.7]
Overnight hospital admission	4.4	[3.2, 6.0]	2.6	[1.9, 3.4]	4.3	[2.6, 7.1]	2.3	[1.8, 3.0]	0.2	[0.1, 0.2]
No consultation with a health professional for mental health	36.3	[33.0, 39.7]	52.0	[49.2, 54.8]	58.1	[52.6, 63.4]	53.4	[51.1, 55.8]	90.0	[89.3, 90.7]

CI: confidence interval. EPC: estimated population count. All estimates exclude respondents with missing data, which ranged from 0.00% to 0.69% (unweighted) per estimate.

a Persons who met criteria for diagnosis of a DSM-IV lifetime affective, anxiety or substance use disorder (with hierarchy) assessed in the survey and had sufficient symptoms of that disorder in the 12 months prior to interview. A person may have more than one class of disorder; therefore, components when added may not add to the total (any 12-month disorder) shown.

b Persons who did not meet criteria for diagnosis of a *DSM*-IV lifetime affective, anxiety or substance use disorder (with hierarchy) assessed in the survey, or who met the criteria but did not have sufficient symptoms of that disorder in the 12 months prior to interview.

c Includes consultation with a general practitioner, psychiatrist, psychologist, other mental health professional or other health professional for mental health, or overnight hospital admission for mental health problems.

d Includes mental health nurse; and other mental health professional. Refer to Table 1, footnote b for further details.

e Includes other health professional; and specialist doctor or surgeon. Refer to Table 1, footnote c for further details.

Relatively more people with 12-month affective disorders consulted a health professional for their mental health (63.7%, 95% CI = [60.3, 67.0]) compared to those with anxiety disorders (48.0%, 95% CI = [45.2, 50.8]) or substance use disorders (41.9%, 95% CI = [36.6, 47.4]). People with affective disorders also consulted GPs and psychologists more commonly than people with anxiety or substance use disorders and consulted psychiatrists more commonly than people with anxiety disorders. Relative to people with disorders, fewer people without disorders consulted any health professional (10.0%, 95% CI = [9.3, 10.7]) or each type of professional.

Between 2007 (Supplementary Tables S2 and S3) and 2020–2022, the proportion of people with 12-month mental disorders who consulted a health professional for their mental health increased by 24%, with greatest increases among those aged 16–24 years (95%), particularly males, and 25–34 years (34%). People with substance use disorders increased their use of any health professional (72%) and GPs (94%). People with anxiety disorders increased their use of GPs (26%) and psychologists (42%). People without disorders increased their use of any health professional (56%), particularly psychologists (250%) and other mental health professionals (111%). Most groups decreased their use of other health professionals.

### Consultation with health professionals for mental health by co-occurring disorder class

Table 4 shows that, when a single disorder class was present, people with affective disorders (52.4%, 95% CI = [46.9, 57.9]) and anxiety disorders (38.6%, 95% CI = [35.1, 42.1]) consulted a health professional for their mental health more commonly than those with substance use disorders (18.3%, 95% CI = [13.4, 24.4]). People with co-occurring disorder classes tended to consult more commonly than those with a single disorder class, although this varied somewhat by type of professional.

**Table 4.** Consultation with health professionals for mental health in the past 12 months, by co-occurring 12-month disorder class (2020–2022).

	Affective disorder only		Anxiety disorder only		Substance use disorder only		Co-occurring affective and anxiety disorders only		Substance use disorders co-occurring with affective and/or anxiety disorders	
	EPC (‘000) = 536.6		EPC (‘000) = 2079.1		EPC (‘000) = 315.0		EPC (‘000) = 773.8		EPC (‘000) = 307.4	
	%	95% CI	%	95% CI	%	95% CI	%	95% CI	%	95% CI
Consultation with any health professional for mental health^a^	52.4	[46.9, 57.9]	38.6	[35.1, 42.1]	18.3	[13.4, 24.4]	67.8	[62.4, 72.7]	66.2	[57.9, 73.6]
General practitioner	40.9	[35.5, 46.6]	29.7	[26.7, 32.9]	13.6	[9.5, 19.2]	58.1	[53.1, 63.1]	52.3	[43.4, 61.1]
Psychiatrist	11.0	[8.0, 14.9]	6.9	[5.6, 8.5]	NA		18.7	[15.5, 22.5]	17.3	[12.0, 24.3]
Psychologist	20.0	[16.0, 24.6]	18.1	[15.5, 21.0]	8.4	[4.9, 14.0]	35.4	[30.1, 41.0]	27.3	[21.0, 34.6]
Other mental health professional^b^	9.8	[6.7, 14.0]	7.0	[5.6, 8.8]	4.4	[2.2, 8.4]	17.3	[13.3, 22.1]	22.5	[15.9, 31.0]
Other health professional^c^	2.4	[0.9, 6.5]	2.8	[2.1, 3.9]	NA		7.1	[4.7, 10.4]	NA	
Overnight hospital admission	NA		1.1	[0.6, 1.9]	NA		4.5	[3.0, 6.7]	8.0	[4.7, 13.2]
No consultation with a health professional for mental health	47.6	[42.1, 53.1]	61.4	[57.9, 64.9]	81.7	[75.6, 86.6]	32.2	[27.3, 37.6]	33.8	[26.4, 42.1]

CI: confidence interval. EPC: estimated population count. NA: not available (cell suppressed due to ABS data safety rules). All estimates exclude respondents with missing data, which ranged from 0.00% to 0.86%. Table includes persons who met criteria for diagnosis of a *DSM*-IV lifetime affective, anxiety or substance use disorder (with hierarchy) assessed in the survey and had sufficient symptoms of that disorder in the 12 months prior to interview.

a Includes consultation with a general practitioner, psychiatrist, psychologist, other mental health professional or other health professional for mental health, or overnight hospital admission for mental health problems.

b Includes mental health nurse, other mental health professionals. Refer to Table 1, footnote b for further details.

c Includes other health professional; and specialist doctor or surgeon. Refer to Table 1, footnote c for further details.

Compared to 2007 (Supplementary Table S4), in 2020–2022 people with anxiety disorders as their only class of disorder were more likely to consult GPs (46%) and less likely to consult other health professionals (−56%).

### Consultation with health professionals for mental health by severity

In the 2020–2022 survey, there was a gradient whereby people with severe 12-month disorders (68.8%, 95% CI = [64.6, 72.7]) consulted a health professional for their mental health more commonly than those with moderate disorders (48.4%, 95% CI = [44.8, 52.0]), who in turn did so more commonly than those with mild disorders (22.9%, 95% CI = [19.6, 26.6]) (Table 5). Consultation with each type of professional generally followed this pattern.

**Table 5.** Consultation with health professionals for mental health in the past 12-months, by severity of 12-month disorder (2020–2022).

	Mild		Moderate		Severe	
	EPC (‘000) = 1110.2		EPC (‘000) = 1874.2		EPC (‘000) = 1032.2	
	%	95% CI	%	95% CI	%	95% CI
Consultation with any health professional for mental health^a^	22.9	[19.6, 26.6]	48.4	[44.8, 52.0]	68.8	[64.6, 72.7]
General practitioner	15.1	[12.6, 18.0]	38.9	[35.6, 42.3]	58.1	[53.8, 62.4]
Psychiatrist	3.9	[2.2, 6.8]	9.0	[7.5, 10.9]	19.8	[17.0, 22.8]
Psychologist	11.0	[8.7, 13.9]	21.0	[18.6, 23.7]	34.2	[30.1, 38.5]
Other mental health professional^b^	3.8	[2.6, 5.5]	9.4	[7.7, 11.4]	19.0	[15.8, 22.7]
Other health professional^c^	1.8	[1.0, 3.1]	3.1	[2.2, 4.4]	6.1	[4.3, 8.7]
Overnight hospital admission	Mild and moderate categories combined:^d^ 0.7 [0.4, 1.3]				6.8	[5.0, 9.2]
No consultation with a health professional for mental health	77.1	[73.4, 80.4]	51.6	[48.0, 55.2]	31.2	[27.3, 35.4]

CI: confidence interval. EPC: estimated population count. All estimates exclude respondents with missing data, which ranged from 0.00% to 0.90% (unweighted) per estimate. Table includes persons who met criteria for diagnosis of a *DSM*-IV lifetime affective, anxiety or substance use disorder (with hierarchy) assessed in the survey and had sufficient symptoms of that disorder in the 12 months prior to interview.

a Includes consultation with a general practitioner, psychiatrist, psychologist, other mental health professional or other health professional for mental health, or overnight hospital admission for mental health problems.

b Includes mental health nurse, other mental health professional. Refer to Table 1, footnote b for further details.

c Includes other health professional; and specialist doctor or surgeon. Refer to Table 1, footnote c for further details.

d Mild and moderate disorders were combined due to low cell counts for these categories individually.

Between 2007 (Supplementary Table S5) and 2020–2022, people with disorders of moderate severity increased their use of GPs (39%), and people with moderate and severe disorders reduced their use of other health professionals (−65% and −56%, respectively).

### Associations with consulting a health professional for mental health

Table 6 presents the results of the logistic regression models that assessed associations with consulting a health professional for mental health in the merged survey data set. After controlling for all characteristics in the model, the odds of consulting any professional, GP, psychologist or other mental health professional, were higher in 2020–2022 than in 2007; the odds of consulting another health professional were lower. Mild, moderate and severe disorders (vs no disorder) were associated with use of any services, GPs and psychologists, whereas moderate and severe disorders were associated with consulting psychiatrists, other mental health professionals and other professionals. Affective disorder and anxiety disorder were positively associated with consulting most types of professionals; substance use disorder was positively associated only with consulting other mental health professionals.

**Table 6.** Factors associated with consultation with health professionals for mental health in the past 12 months.

	Any health professional^a^		General practitioner		Psychiatrist		Psychologist		Other mental health professional^b^		Other health professional^c^	
	aOR	95% CI	aOR	95% CI	aOR	95% CI	aOR	95% CI	aOR	95% CI	aOR	95% CI
Survey												
2020–2022	1.48	[1.30, 1.68]	1.50	[1.31, 1.72]	1.23	[0.88, 1.72]	2.11	[1.76, 2.53]	1.56	[1.23, 1.97]	0.42	[0.32, 0.55]
2007	1.00		1.00		1.00		1.00		1.00		1.00	
Wald χ^2^ (*p*)	37.10	<0.001	34.52	<0.001	1.48	0.223	65.72	<0.001	13.81	<0.001	40.70	<0.001
Severity												
None (no 12-month disorder^d^)	1.00		1.00		1.00		1.00		1.00		1.00	
Mild	1.65	[1.24, 2.20]	1.87	[1.40, 2.50]	1.88	[0.95, 3.72]	2.15	[1.47, 3.15]	1.36	[0.80, 2.30]	1.11	[0.53, 2.32]
Moderate	3.74	[2.80, 4.99]	4.60	[3.45, 6.15]	3.51	[2.17, 5.69]	3.32	[2.31, 4.77]	2.59	[1.60, 4.18]	2.51	[1.14, 5.49]
Severe	6.98	[4.74, 10.28]	8.57	[5.98, 12.27]	7.11	[4.07, 12.41]	5.30	[3.31, 8.48]	5.67	[3.16, 10.17]	3.46	[1.70, 7.05]
Wald χ^2^ (*p*)	125.44	<0.001	160.21	<0.001	61.73	<0.001	52.11	<0.001	46.96	<0.001	21.96	<0.001
Any 12-month affective disorder												
No	1.00		1.00		1.00		1.00		1.00		1.00	
Yes	2.59	[2.06, 3.25]	2.26	[1.83, 2.79]	1.81	[1.31, 2.51]	1.90	[1.42, 2.54]	1.81	[1.28, 2.55]	1.74	[1.16, 2.63]
Wald χ^2^ (*p*)	69.06	<0.001	59.86	<0.001	12.94	<0.001	19.15	<0.001	11.81	0.001	7.20	0.007
Any 12-month anxiety disorder												
No	1.00		1.00		1.00		1.00		1.00		1.00	
Yes	1.74	[1.38, 2.19]	1.53	[1.20, 1.95]	1.58	[1.07, 2.32]	1.69	[1.26, 2.26]	1.43	[0.94, 2.16]	2.30	[1.23, 4.31]
Wald χ^2^ (*p*)	22.54	<0.001	11.79	0.001	5.52	0.019	12.58	<0.001	2.88	0.090	6.95	0.008
Any 12-month substance use disorder												
No	1.00		1.00		1.00		1.00		1.00		1.00	
Yes	1.10	[0.82, 1.46]	1.04	[0.80, 1.35]	1.08	[0.71, 1.65]	0.92	[0.65, 1.31]	1.84	[1.21, 2.80]	1.18	[0.66, 2.12]
Wald χ^2^ (*p*)	0.40	0.529	0.09	0.767	0.13	0.722	0.21	0.646	8.41	0.004	0.34	0.562
Sex												
Male	1.00		1.00		1.00		1.00		1.00		1.00	
Female	1.69	[1.49, 1.91]	1.80	[1.54, 2.09]	1.00	[0.76, 1.31]	1.40	[1.20, 1.64]	1.70	[1.35, 2.15]	1.70	[1.18, 2.46]
Wald χ^2^ (*p*)	70.68	<0.001	58.66	<0.001	0.00	0.984	18.32	<0.001	20.32	<0.001	8.14	0.004
Age group at survey (years)												
16–25	1.57	[1.30, 1.90]	1.35	[1.05, 1.73]	0.79	[0.49, 1.26]	2.77	[1.92, 3.99]	3.28	[2.07, 5.19]	0.80	[0.42, 1.52]
26–45	2.39	[1.99, 2.88]	2.41	[1.96, 2.95]	1.40	[0.96, 2.05]	3.54	[2.55, 4.90]	3.40	[2.26, 5.12]	1.73	[1.02, 2.93]
46–65	1.89	[1.61, 2.23]	1.85	[1.52, 2.25]	1.31	[0.92, 1.87]	2.24	[1.59, 3.15]	2.58	[1.69, 3.92]	1.38	[0.77, 2.47]
66–85	1.00		1.00		1.00		1.00		1.00		1.00	
Wald χ^2^ (*p*)	104.52	<0.001	97.42	<0.001	13.76	0.003	62.91	<0.001	35.12	<0.001	17.41	0.001
Marital status												
Married	1.00		1.00		1.00		1.00		1.00		1.00	
Previously married	1.58	[1.34, 1.88]	1.44	[1.20, 1.73]	0.97	[0.69, 1.36]	1.67	[1.32, 2.11]	2.32	[1.67, 3.21]	1.30	[0.84, 2.03]
Never married	1.33	[1.15, 1.53]	1.31	[1.11, 1.56]	1.54	[1.13, 2.10]	1.36	[1.12, 1.66]	1.54	[1.17, 2.03]	1.45	[0.98, 2.15]
Wald χ^2^ (*p*)	32.10	<0.001	19.06	<0.001	10.65	0.005	20.59	<0.001	28.74	<0.001	3.74	0.154
Personal income												
Quintiles 1–2 (lower)	1.01	[0.83, 1.22]	1.15	[0.93, 1.41]	1.57	[1.09, 2.26]	0.79	[0.62, 1.00]	1.06	[0.76, 1.47]	0.92	[0.59, 1.46]
Quintiles 3–4	0.85	[0.71, 1.01]	1.05	[0.83, 1.32]	1.15	[0.80, 1.66]	0.70	[0.56, 0.87]	0.93	[0.68, 1.29]	0.79	[0.51, 1.23]
Quintile 5 (high)	1.00		1.00		1.00		1.00		1.00		1.00	
Missing information	0.75	[0.60, 0.95]	0.82	[0.63, 1.06]	1.05	[0.63, 1.76]	0.63	[0.46, 0.86]	1.01	[0.65, 1.57]	1.41	[0.87, 2.29]
Wald χ^2^ (*p*)	12.46	0.006	7.48	0.058	10.73	0.013	14.14	0.003	1.04	0.791	5.40	0.145
Index of relative socioeconomic disadvantage (IRSD)^e^												
Quintiles 1–2 (greater disadvantage)	0.84	[0.73, 0.98]	0.83	[0.71, 0.96]	0.73	[0.54, 0.99]	0.78	[0.62, 0.98]	1.09	[0.81, 1.47]	0.65	[0.44, 0.97]
Quintiles 3–4	0.96	[0.83, 1.09]	0.92	[0.78, 1.07]	0.74	[0.54, 1.02]	0.90	[0.75, 1.08]	1.24	[0.91, 1.69]	0.86	[0.61, 1.23]
Quintile 5 (least disadvantage)	1.00		1.00		1.00		1.00		1.00		1.00	
Wald χ^2^ (*p*)	5.97	0.051	6.19	0.045	4.48	0.107	4.52	0.104	2.00	0.367	4.72	0.095
Remoteness area^f^												
Major cities	1.00		1.00		1.00		1.00		1.00		1.00	
Inner regional	1.11	[0.93, 1.31]	1.20	[0.99, 1.45]	0.89	[0.60, 1.32]	0.72	[0.58, 0.89]	1.23	[0.89, 1.68]	1.10	[0.79, 1.52]
Other areas, including outer regional and remote	0.87	[0.70, 1.08]	0.97	[0.77, 1.21]	0.79	[0.47, 1.32]	0.62	[0.47, 0.82]	1.32	[0.94, 1.84]	0.97	[0.57, 1.65]
Wald χ^2^ (*p*)	3.21	0.201	3.96	0.138	0.98	0.613	18.31	<0.001	3.67	0.160	0.36	0.837

aOR: adjusted odds ratio. CI: confidence interval. Of the 24,732 respondents in the analysis sample, 8.7% (unweighted) had missing data on personal income, so this was retained as a category in the personal income variable. Regression models excluded respondents with missing data on any variable other than personal income (0.02%–0.10% missing per model).

a Includes consultation with a general practitioner, psychiatrist, psychologist, other mental health professional or other health professional for mental health, or overnight hospital admission for mental health problems.

b In 2020–2022, includes mental health nurse; and other mental health professional (refer to Table 1, footnote b for further details). In 2007, includes mental health nurse; and other professional providing specialist mental health services (refer to Supplementary Table S1, footnote b for further details).

c In 2020–2022, includes other health professional; and specialist doctor or surgeon (refer to Table 1, footnote c for further details). In 2007, includes other professional providing general services; specialist doctor or surgeon; and complementary/alternative therapist (refer to Supplementary Table S1, footnote c for further details).

d Persons who did not meet criteria for diagnosis of a *DSM*-IV lifetime affective, anxiety or substance use disorder (with hierarchy) assessed in the survey, or who met the criteria but did not have sufficient symptoms of that disorder in the 12 months prior to interview.

e The IRSD summarises information from a range of measures of the economic and social disadvantage of people and households within an area. A low score indicates relatively greater disadvantage (ABS, 2018b).

f Remoteness area is an area-level measure of relative geographic access to services (ABS, 2007, 2018a).

Males (vs females) had lower odds of consulting all types of professionals except psychiatrists. People aged 66–85 years had lower odds of consulting any health professional, GP, psychologist or other mental health professional, than all younger age groups. Being previously or never married tended to be positively associated with consulting most types of professionals (except other health professionals). Compared to people with a high level of income (quintile 5), people with lower levels of income (quintiles 1-2) had higher odds of consulting a psychiatrist, people with intermediate levels of income (quintiles 3-4) had lower odds of consulting a psychologist, and people with missing income information had lower odds of consulting a GP or psychologist. Compared to people living in areas of least disadvantage, those living in areas of relatively greater disadvantage had lower odds of consulting a GP. Compared to people living in major cities, people living in regional and remote areas had lower odds of consulting a psychologist.

**Figure 1.**
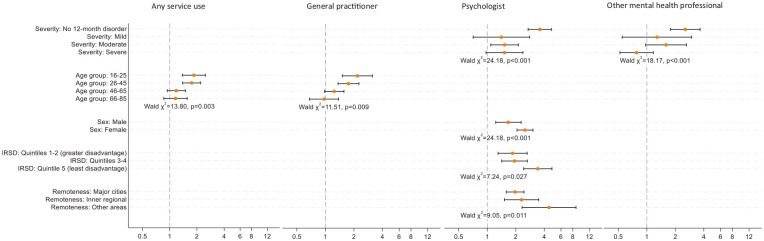
Results of subgroup analyses showing the odds of service use for mental health problems in 2020–2022 compared to 2007. IRSD: Index of Relative Socioeconomic Disadvantage. Results shown only for variables with evidence of interaction with survey year (*p* < 0.05). Figure displays adjusted odds ratios and 95% CIs. For age group, sex at birth, IRSD and Remoteness, odds ratios are adjusted for all other variables in the multivariate models. For severity, odds ratios are adjusted for all other variables in the multivariate models except severity, 12-month affective disorder, 12-month anxiety disorder and 12-month substance use disorder.

Interaction analyses indicated changes in the odds of consultation over time for some population subgroups (see Figure 1). Notably, people aged 16–45 years had approximately twice the odds of consulting any health professional and GPs in 2020–2022 than their counterparts in 2007, whereas there was little evidence of change for older age groups. People without disorders or with disorders of moderate severity had higher odds of consulting a psychologist in 2020–2022 than in 2007, and people without disorders had higher odds of consulting another mental health professional in 2020–2022 than in 2007. The odds of consultation with psychologists increased between 2007 and 2020–2022 for all subgroups of sex, relative socioeconomic disadvantage and remoteness; however, the extent of increase was greatest for females, people living in less disadvantaged areas, and people living in outer regional and remote areas.

## Discussion

Our study revealed several important findings regarding Australians’ use of health professionals for mental health. A key finding was that, in 2020–2022, 47% of people with 12-month disorders had consulted a health professional for mental health or had an overnight admission for mental health problems in the past year, with a gradient according to severity of disorder (mild 23%, moderate 48% and severe 69%). This represents a meaningful overall increase from 38% in 2007 but falls short of the targets based on ‘desirable’ service demand proposed in the National Mental Health Service Planning Framework: overall 67%, mild 50%, moderate 80% and severe 100% (AIHW, 2022).

Nearly one-third of people with severe disorders had not consulted a health professional for their mental health in the past year, essentially unchanged from 2007. There are several possible reasons for this lack of change. Newer service models might not yet have impacted treatment rates (e.g. Adult Mental Health Centres have been progressively implemented since 2020) or target only a minority of people with severe problems (e.g. NDIS-funded psychosocial support services are limited to people with permanent impairment, aged below 65 years). Meanwhile, the rate of persons treated in extant public sector specialised mental health services increased only marginally (AIHW, 2023b). Reduced provider capacity and rising out-of-pocket costs may have limited opportunities to consult mental health specialists in private practice (Callander, 2023; Neil, 2023; Pirkis et al., 2022). Challenges in navigating the mental health service system, and structural stigma and discrimination, may be persistent barriers for this group (Dixon et al., 2016; Fagiolini and Goracci, 2009; Reavley and Morgan, 2021). Our results support the continued development of service models to meet the diverse needs of people with severe disorders (Department of Health and Aged Care, 2015; National Mental Health Commission, 2014; Productivity Commission, 2020).

People with substance use disorders as their *only* class of disorder continued to have low rates of consultation with health professionals for their mental health. Preventive screening (e.g. in primary care, emergency departments) and public education to increase problem recognition and awareness of treatment benefits could help to improve perceived need for treatment which is typically low in this group (Lubman et al., 2007; Mojtabai and Crum, 2013). In contrast, the rates of consultation among people with substance use disorders co-occurring with affective and/or anxiety disorders were comparable to people with co-occurring affective and anxiety disorders only, whereas they had been lower in 2007. This might reflect a shift in the mix of co-occurring disorders among those with substance use disorders. It might also reflect the impact of recent clinical and policy guidance regarding the management of co-occurring substance use disorders (e.g. Marel et al., 2022; Mills et al., 2019; NSW Department of Health, 2008; Queensland Health, 2021).

The observed increases in consultation with health professionals for mental health in 2020–2022 (vs 2007) were not experienced uniformly in the population. For example, our subgroup analyses showed two- to threefold increases in consultation with psychologists and other mental health professionals by people without 12-month disorders (vs comparatively less change among people with disorders). Many of these people are likely receiving services funded through Better Access, which is currently the main way that Australians receive psychology services (Pirkis et al., 2022); however, some with lower levels of need could benefit from low-intensity service options that require little or no involvement from a mental health specialist (AIHW, 2022; National Mental Health Commission, 2014). Our findings highlight the need for tools to effectively match treatment to need and to prioritise higher-intensity services for people with moderate and severe illness (Thornicroft, 2007).

Despite an increase in consultation with health professionals for mental health among younger adult males with disorders, females were generally more likely to consult (except psychiatrists) than males. The greater likelihood of consulting psychologists by females (vs males) became more pronounced over time. Continued efforts to address barriers to help-seeking among males are indicated.

In our multivariate regression analyses controlling for a range of clinical and other characteristics, people aged 66–85 years were less likely than all younger age groups to consult any health professional and to consult GPs, psychologists and other mental health professionals. Some older people may view mental ill-health as a normal consequence of ageing and therefore not perceive a need for treatment (AIFS, 2019; Askari et al., 2023). Consumer and practitioner beliefs that psychological therapies are less effective for older adults may also play a part (Maust et al., 2015; Saunders et al., 2021). These findings support calls for benchmarks and standards to guide the availability and quality of care for older Australians (Hariharan, n.d.; The Royal Australian and New Zealand College of Psychiatrists, 2019).

In contrast, the gap in health professional consultation between younger and middle-aged adults appeared to have narrowed over time. Other analyses of the 2020–2022 and 2007 NSMHWBs show increases in 12-month disorder prevalence and co-occurrence among younger adults (Slade et al., 2024; Sunderland et al., 2024). Importantly, our descriptive analyses (Table 2 and Supplementary Table S2) showed increased health professional consultation *within* the subset of younger adults with disorders, suggesting that increased consultation at a population level was not solely due to increased disorder prevalence. Younger adults consulted GPs more commonly in 2020–2022 than in 2007, consistent with other evidence of increased use of major primary mental health care programmes by young people (Harris et al., 2010; headspace, 2019, 2021; Pirkis et al., 2022).

In our multivariate analyses, we found that different socioeconomic and geographic characteristics were associated with consulting different types of providers. High-income earners and city dwellers were more likely to consult psychologists, likely reflecting the concentration of psychologists in more urban and affluent areas and increasing consumer co-payments for private psychology services (Pirkis et al., 2022; Rosenberg et al., 2022). That said, people living in outer regional/remote areas had a more pronounced *increase* in the use of psychologists between 2007 and 2020–2022, possibly due to the widespread implementation of telehealth psychology services funded through Medicare from 2020. People living in relatively more disadvantaged areas were less likely to consult a GP for mental health, perhaps because they were less likely to have long GP consultations and therefore less opportunity to discuss mental health needs, or avoided consulting a GP due to cost (ABS, 2023b; Furler et al., 2002). Interestingly, having a lower income was associated with seeing a psychiatrist. Although psychiatrists are concentrated in more affluent areas (AIHW, 2023c), they tend to see people with relatively more severe and complex problems who, on average, have lower incomes (Enticott et al., 2022; Isaacs et al., 2018; Pulkki-Råback et al., 2012) and they more often work in publicly funded services (AIHW, 2023c). Our findings underscore the need to strengthen the mental health workforce in underserved areas and ensure that consumer co-payments are affordable (Cleary et al., 2020; Department of Health and Aged Care, 2022c; Medicare Benefits Schedule Review Taskforce, 2020).

### Study limitations

As described in the ‘Method’ section, the examples of professionals included in the ‘other health professionals’ category differed between the surveys. This could have contributed to the lower rates of consulting other health professionals in the 2020–2022 survey. Respondents’ reports of service use were not externally validated, although sex- and provider-specific estimates from the 2020–2022 survey were comparable to those reported from a larger, contemporaneous health survey (ABS, 2021), lending some support for their validity. The consultation data analysed in our study were not disorder-specific, so we do not know whether consulting a given professional was related to a particular mental disorder.

The surveys did not assess very low prevalence or difficult to assess disorders (e.g. schizophrenia, personality disorders); these would require a larger sample and skilled interviewers to assess reliably. The response rate was relatively low in both surveys; this could affect the validity of estimates as non-response can vary across respondent characteristics (ABS, 2008). Relatively more non-response in 2020–2022 was due to difficulties in contacting participants and completing interviews and less was due to refusal, compared to 2007 (ABS, 2008, 2023a).

While we commented on whether our findings might reflect the impacts of recent service reforms, other trends or shocks including the COVID-19 pandemic might also have played a part. The 2020–2022 NSMHWB was conducted in two cohorts due to COVID-19-related lockdowns; it may be that mental health and associated service use differed between these cohorts due to the impacts of the pandemic. The ABS (2023a) reports small differences in prevalence of *DSM*-IV mental disorders between the 2020–2022 NSMHWB cohorts (20.9% in Cohort 1 vs 19.7% in Cohort 2). However, other sources indicate that psychological distress and use of some mental health services (e.g. claims for Medicare-subsidised mental health services and mental health-related prescriptions) have increased in Australia since the onset of the COVID-19 pandemic (AIHW, 2024; Ellis et al., 2022). Disentangling pandemic effects from background trends in prevalence and service use, the impact of prior and pandemic-era policy and service reforms, and other societal and environmental changes will require careful modelling (Botha et al., 2023; Skinner et al., 2024). Our study was designed to provide a high-level overview of patterns of consultation with health professionals for mental health; future studies investigating characteristics of these consultations (e.g. types of interventions received, number of visits, modality of delivery) may provide insights into the nature and quality of care delivered. Future studies could examine the use of other services (e.g. digital services not involving a health professional) and self-management strategies that people use to manage their mental health, who uses them, and how they intersect with the use of health professionals. Income and relative socioeconomic disadvantage were organised into broad groups; patterns could be more nuanced.

## Conclusion

Relatively more Australian adults consulted a health professional for their mental health in 2020–2022 than in 2007, but proportions remain below national targets. Of particular concern, consultation among people with severe disorders did not increase. While some differentials in consulting appear to have decreased over time (namely, use of psychologists by people in outer regional/remote areas, and use of GPs by younger adults), others increased (namely, use of psychologists by people without 12-month disorders and people in the least disadvantaged areas). Efforts remain needed to encourage people with lower levels of need to use low-intensity services when appropriate and to ensure that people with higher levels of need have access to necessary specialist clinical care and supports. Persistently lower rates of service use among people with substance use disorders as their only disorder, older Australians and males, and socioeconomic differentials in access to some types of professionals, require attention.

## Supplemental Material

sj-docx-1-anp-10.1177_00048674241307919 – Supplemental material for Consultation with health professionals for mental health in Australia in 2020–2022 and changes since 2007: Findings from the 2020–2022 National Study of Mental Health and Wellbeing Supplemental material, sj-docx-1-anp-10.1177_00048674241307919 for Consultation with health professionals for mental health in Australia in 2020–2022 and changes since 2007: Findings from the 2020–2022 National Study of Mental Health and Wellbeing by Meredith G Harris, Caley Tapp, Joshua J Vescovi, Matthew Sunderland, Sandra Diminic, Cath Chapman, Tim N Slade, Maree Teesson, Jane Pirkis and Philip M Burgess in Australian & New Zealand Journal of Psychiatry
