# Supplementary material for: Consultation with health professionals for mental health in Australia in 2020–2022 and changes since 2007: Findings from the 2020–2022 National Study of Mental Health and Wellbeing
Source: Aust N Z J Psychiatry. 2025 Jul 15;59(9):810–23. doi: 10.1177/00048674241307919 (PMC12397540; doi:10.1177/00048674241307919)
Supplement: sj-docx-1-anp-10.1177_00048674241307919 – Supplemental material for Consultation with health professionals for mental health in Australia in 2020–2022 and changes since 2007: Findings from the 2020–2022 National Study of Mental Health and Wellbeing [file sj-docx-1-anp-10.1177_00048674241307919.docx]

**Table S1.** Consultation with health professionals for mental health in the past 12-months, by sex (2007)

|  | **Males** | | | **Females** | | | **Persons** | | |
| --- | --- | --- | --- | --- | --- | --- | --- | --- | --- |
|  | EPC ('000) | % | 95% CI | EPC ('000) | % | 95% CI | EPC ('000) | % | 95% CI |
| **Consultation with any health professional for mental health** ^a^ | 699.9 | 8.8 | (7.5-10.3) | 1200.9 | 14.9 | (13.6-16.3) | 1900.8 | 11.9 | (11.0-12.8) |
| **General practitioner** | 465.3 | 5.9 | (4.8-7.2) | 824.2 | 10.2 | (9.2-11.3) | 1289.5 | 8.1 | (7.4-8.8) |
| **Psychiatrist** | 155.5 | 2.0 | (1.3-2.9) | 211.7 | 2.6 | (1.9-3.6) | 367.2 | 2.3 | (1.8-3.0) |
| **Psychologist** | 250.8 | 3.2 | (2.5-4.0) | 314.7 | 3.9 | (3.3-4.5) | 565.6 | 3.5 | (3.1-4.1) |
| **Other mental health professional** ^b^ | 126.0 | 1.6 | (1.1-2.3) | 230.0 | 2.9 | (2.4-3.4) | 356.1 | 2.2 | (1.9-2.7) |
| **Other health professional** ^c^ | 122.4 | 1.5 | (1.0-2.3) | 262.7 | 3.3 | (2.6-4.1) | 385.0 | 2.4 | (2.0-2.9) |
| **Overnight hospital admission** | 66.8 | 0.8 | (0.5-1.5) | 49.1 | 0.6 | (0.4-0.9) | 116.0 | 0.7 | (0.5-1.1) |
| **No consultation with a health professional for mental health** | 7241.1 | 91.2 | (89.7-92.5) | 6863.4 | 85.1 | (83.7-86.4) | 14104.4 | 88.1 | (87.2-89.0) |
| CI: confidence interval. EPC: estimated population count. All estimates exclude respondents with missing data, which ranged from 0.00% to 0.07% (unweighted) per estimate. Analysis approach builds on previous work by Burgess et al. (2009; Table 1). | | | | | | | | | |
| ^a^ Includes consultation with a general practitioner, psychiatrist, psychologist, other mental health professional or other health professional for mental health, or overnight hospital admission for mental health problems. | | | | | | | | | |
| ^b^ Includes mental health nurse, other professional providing specialist mental health services (including social worker, counsellor, occupational therapist). | | | | | | | | | |
| ^c^ Includes other professional providing general services (including social worker, occupational therapist, counsellor); specialist doctor or surgeon (including cardiologist, gynaecologist, or urologist); and complementary/alternative therapist (such as herbalist or naturopath). | | | | | | | | | |

**Table S2.** Consultation with health professionals for mental health in the past 12-months among people with any 12-month disorder, by age group and sex (2007)

| **Age group (years)** | **Males** | | | **Females** | | | **Persons** | | |
| --- | --- | --- | --- | --- | --- | --- | --- | --- | --- |
|  | EPC ('000) | % | 95% CI | EPC ('000) | % | 95% CI | EPC ('000) | % | 95% CI |
| **16-24** | 36.6 | 13.6 | (7.9-22.5) | 106.0 | 32.4 | (25.3-40.5) | 142.6 | 23.9 | (18.8-30.0) |
| **25-34** | 80.5 | 28.3 | (19.6-39.0) | 149.7 | 46.7 | (38.1-55.5) | 230.3 | 38.1 | (31.5-45.1) |
| **35-44** | 106.1 | 37.0 | (26.7-48.6) | 176.4 | 51.0 | (39.3-62.7) | 282.5 | 44.7 | (38.1-51.4) |
| **45-54** | 103.2 | 42.6 | (27.0-59.9) | 124.9 | 40.7 | (27.8-55.1) | 228.1 | 41.6 | (30.0-54.1) |
| **55-64** | 44.8 | 37.1 | (24.0-52.4) | 77.1 | 43.7 | (32.8-55.2) | 121.8 | 41.0 | (32.5-50.1) |
| **65-85** | 17.5 | 26.2 | (14.9-41.9) | 38.5 | 46.8 | (32.6-61.4) | 56.1 | 37.6 | (27.9-48.3) |
| **Total** | 388.7 | 30.6 | (25.2-36.6) | 672.7 | 43.1 | (38.2-48.2) | 1061.4 | 37.5 | (34.1-41.1) |
| CI: confidence interval. EPC: estimated population count. Analysis approach builds on previous work by Burgess et al. (2009; Table 2). Consultation with health professionals includes consultation with a general practitioner, psychiatrist, psychologist, other mental health professional or other health professional for mental health, or overnight hospital admission for mental health problems. Table includes persons who met criteria for diagnosis of a DSM-IV lifetime affective, anxiety or substance use disorder (with hierarchy) assessed in the survey and had sufficient symptoms of that disorder in the 12 months prior to interview. | | | | | | | | | |

**Table S3.** Consultation with health professionals for mental health in the past 12-months, by 12-month disorder class (2007)

|  | **Any 12-month affective disorder** | | **Any 12-month anxiety disorder** | | **Any 12-month substance use disorder** | | **Any 12-month disorder** ^a^ | | **No 12-month disorder ^b^** | |
| --- | --- | --- | --- | --- | --- | --- | --- | --- | --- | --- |
|  | EPC ('000) = 933.1 | | EPC ('000) = 1884.3 | | EPC ('000) = 811.3 | | EPC ('000) = 2828.9 | | EPC ('000) = 13186.4 | |
|  | % | 95% CI | % | 95% CI | % | 95% CI | % | 95% CI | % | 95% CI |
| **Consultation with any health professional for mental health ^c^** | 60.9 | (54.1-67.2) | 42.6 | (38.3-47.1) | 24.4 | (18.6-31.4) | 37.5 | (34.1-41.1) | 6.4 | (5.6-7.3) |
| **General practitioner** | 47.2 | (41.5-53.1) | 30.6 | (26.7-34.7) | 16.9 | (12.7-22.1) | 27.1 | (24.4-29.9) | 4.0 | (3.4-4.7) |
| **Psychiatrist** | 13.6 | (9.6-18.9) | 9.9 | (6.7-14.5) | 5.8 | (3.7-9.1) | 8.1 | (5.8-11.3) | 1.0 | (0.7-1.5) |
| **Psychologist** | 23.9 | (19.2-29.3) | 16.4 | (13.1-20.2) | 9.4 | (6.3-13.9) | 14.3 | (12.0-17.0) | 1.2 | (1.0-1.6) |
| **Other mental health professional** ^d^ | 16.2 | (11.9-21.8) | 9.5 | (7.2-12.3) | 9.0 | (5.5-14.5) | 8.6 | (6.9-10.6) | 0.9 | (0.7-1.1) |
| **Other health professional** ^e^ | 11.9 | (8.5-16.5) | 9.1 | (7.2-11.6) | 5.7 | (3.2-9.9) | 7.4 | (5.9-9.4) | 1.3 | (1.0-1.8) |
| **Overnight hospital admission** | 6.5 | (3.8-10.8) | 3.2 | (2.0-5.2) | 4.2 | (2.0-8.4) | 2.7 | (1.7-4.2) | 0.3 | (0.2-0.6) |
| **No consultation with a health professional for mental health** | 39.1 | (32.8-45.9) | 57.4 | (52.9-61.7) | 75.6 | (68.6-81.4) | 62.5 | (58.9-65.9) | 93.6 | (92.7-94.4) |
| CI: confidence interval. EPC, estimated population count. All estimates exclude respondents with missing data, which ranged from 0.00% to 0.48% (unweighted) per estimate. Analysis approach builds on previous work by Burgess et al. (2009; Table 3). | | | | | | | | | | |
| ^a^ Persons who met criteria for diagnosis of a DSM-IV lifetime affective, anxiety or substance use disorder (with hierarchy) assessed in the survey and had sufficient symptoms of that disorder in the 12 months prior to interview. A person may have more than one class of disorder, therefore components when added may not add to the total (any 12-month disorder) shown. | | | | | | | | | | |
| ^b^ Persons who did not meet criteria for diagnosis of a DSM-IV lifetime affective, anxiety or substance use disorder (with hierarchy) assessed in the survey, or who met the criteria but did not have sufficient symptoms of that disorder in the 12 months prior to interview. | | | | | | | | | | |
| ^c^ Includes consultation with a general practitioner, psychiatrist, psychologist, other mental health professional or other health professional for mental health, or overnight hospital admission for mental health problems. | | | | | | | | | | |
| ^d^ Includes: mental health nurse, other professional providing specialist mental health services. Refer to Supplementary Table S1, footnote b for further detail. | | | | | | | | | | |
| ^e^ Includes: other professional providing general services; specialist doctor or surgeon; and complementary/alternative therapist. Refer to Supplementary Table S1, footnote c for further detail. | | | | | | | | | | |

**Table S4.** Consultation with health professionals for mental health in the past 12-months, by co-occurring 12-month disorder class (2007)

|  | **Affective disorder only** | | **Anxiety disorder only** | | **Substance use disorder only** | | **Co-occurring affective and anxiety disorders only** | | **Substance use disorders co-occurring with affective and/or anxiety disorders** | |
| --- | --- | --- | --- | --- | --- | --- | --- | --- | --- | --- |
|  | EPC ('000) = 386.4 | | EPC ('000) = 1219.6 | | EPC ('000) = 531.8 | | EPC ('000) = 411.7 | | EPC ('000) = 279.5 | |
|  | % | 95% CI | % | 95% CI | % | 95% CI | % | 95% CI | % | 95% CI |
| **Consultation with any health professional for mental health** ^a^ | 45.7 | (36.3-55.3) | 31.7 | (26.7-37.2) | 12.3 | (8.0-18.4) | 72.8 | (64.1-80.0) | 47.5 | (35.9-59.4) |
| **General practitioner** | 35.1 | (26.9-44.4) | 20.4 | (16.6-25.0) | 8.2 | (5.4-12.1) | 59.1 | (50.7-66.9) | 33.7 | (23.8-45.3) |
| **Psychiatrist** | 6.0 | (2.6-13.4) | 6.2 | (3.1-12.1) | 3.6 | (1.8-7.3) | 20.2 | (14.0-28.2) | 10.0 | (5.1-18.8) |
| **Psychologist** | 16.7 | (10.4-25.8) | 11.3 | (7.9-15.8) | 4.4 | (2.0-9.6) | 30.6 | (23.4-38.9) | 19.0 | (11.5-29.6) |
| **Other mental health professional** ^b^ | 11.0 | (6.1-19.0) | 4.4 | (3.1-6.4) | 4.0 | (1.9-8.2) | 17.6 | (12.3-24.5) | 18.5 | (10.4-30.8) |
| **Other health professional** ^c^ | 7.5 | (3.4-15.8) | 6.3 | (4.3-9.1) | NA |  | 14.0 | (9.4-20.4) | 13.9 | (7.2-25.1) |
| **Overnight hospital admission** | NA |  | NA |  | NA |  | 5.5 | (3.2-9.2) | 11.2 | (5.3-22.1) |
| **No consultation with a health professional for mental health** | 54.3 | (44.7-63.7) | 68.3 | (62.8-73.3) | 87.7 | (81.6-92.0) | 27.2 | (20.0-35.9) | 52.5 | (40.6-64.1) |
| CI: confidence interval. EPC: estimated population count. NA: not available (cell suppressed due to ABS data safety rules). All estimates exclude respondents with missing data, which ranged from 0.00% to 1.38% (unweighted) per estimate. Table includes persons who met criteria for diagnosis of a DSM-IV lifetime affective, anxiety or substance use disorder (with hierarchy) assessed in the survey and had sufficient symptoms of that disorder in the 12 months prior to interview. Analysis approach builds on previous work by Burgess et al. (2009; Table 4). | | | | | | | | | | |
| ^a^ Includes consultation with a general practitioner, psychiatrist, psychologist, other mental health professional or other health professional for mental health, or overnight hospital admission for mental health problems. | | | | | | | | | | |
| ^b^ Includes: mental health nurse; and other professional providing specialist mental health services. Refer to Supplementary Table S1, footnote b for further detail. | | | | | | | | | | |
| ^c^ Includes: other professional providing general services; specialist doctor or surgeon; and complementary/alternative therapist. Refer to Supplementary Table S1, footnote c for further detail. | | | | | | | | | | |

**Table S5.** Consultation with health professionals for mental health in the past 12-months, by severity of 12-month disorder (2007)

|  | **Mild** | | **Moderate** | | **Severe** | |
| --- | --- | --- | --- | --- | --- | --- |
|  | EPC ('000) = 1146.2 | | EPC ('000) = 1099.2 | | EPC ('000) = 589.9 | |
|  | % | 95% CI | % | 95% CI | % | 95% CI |
| **Consultation with any health professional for mental health** ^a^ | 18.7 | (14.4-23.9) | 41.0 | (35.9-46.3) | 68.2 | (60.1-75.3) |
| **General practitioner** | 12.2 | (9.3-15.8) | 27.9 | (23.7-32.4) | 55.4 | (48.3-62.4) |
| **Psychiatrist** | 3.5 | (1.5-8.1) | 7.3 | (4.5-11.5) | 18.8 | (13.7-25.3) |
| **Psychologist** | 7.9 | (4.8-12.7) | 14.6 | (11.4-18.6) | 26.5 | (20.3-33.8) |
| **Other mental health professional** ^b^ | 3.1 | (1.7-5.5) | 6.2 | (4.4-8.6) | 24.1 | (18.3-30.9) |
| **Other health professional** ^c^ | 2.6 | (1.6-4.0) | 8.9 | (6.2-12.6) | 14.0 | (9.6-20.1) |
| **Overnight hospital admission** | NA |  | NA |  | NA |  |
| **No consultation with a health professional for mental health** | 81.3 | (76.1-85.6) | 59.0 | (53.7-64.1) | 31.8 | (24.7-39.9) |
| CI: confidence interval. EPC: estimated population count. NA: not available (cell suppressed due to ABS data safety rules). All estimates exclude respondents with missing data, which ranged from 0.00% to 1.14% (unweighted) per estimate. Table includes persons who met criteria for diagnosis of a DSM-IV lifetime affective, anxiety or substance use disorder (with hierarchy) assessed in the survey and had sufficient symptoms of that disorder in the 12 months prior to interview. Analysis approach builds on previous work by Burgess et al. (2009; Table 5). | | | | | | |
| ^a^ Includes consultation with a general practitioner, psychiatrist, psychologist, other mental health professional or other health professional for mental health, or overnight hospital admission for mental health problems. | | | | | | |
| ^b^ Includes: mental health nurse; and other professional providing specialist mental health services. Refer to Supplementary Table S1, footnote b for further detail. | | | | | | |
| ^c^ Includes: other professional providing general services; specialist doctor or surgeon; and complementary/alternative therapist. Refer to Supplementary Table S1, footnote c for further detail. | | | | | | |

**References:**

Burgess PM, Pirkis JE, Slade TN, et al. (2009) Service use for mental health problems: Findings from the 2007 National Survey of Mental Health and Wellbeing. *Australian & New Zealand Journal of Psychiatry* 43(7): 615-623.
